# Structuring effects of chemicals from the sea fan *Phyllogorgia dilatata* on benthic communities

**DOI:** 10.7717/peerj.3186

**Published:** 2017-04-04

**Authors:** Felipe V. Ribeiro, Bernardo A.P. da Gama, Renato C. Pereira

**Affiliations:** Departamento de Biologia Marinha, Universidade Federal Fluminense, Niterói, Rio de Janeiro, Brasil

**Keywords:** Secondary metabolites, *Phyllogorgia dilatata*, Gorgonian, Marine chemical ecology, Octocoral, Allelopathy, Community effects, Benthic ecology, Rocky reefs

## Abstract

Despite advances in understanding the ecological functions of secondary metabolites from marine organisms, there has been little focus on the influence of chemically-defended species at the community level. Several compounds have been isolated from the gorgonian octocoral *Phyllogorgia dilatata*, a conspicuous species that forms dense canopies on rocky reefs of northern Rio de Janeiro State, Brazil. Manipulative experiments were performed to study: (1) the effects of live colonies of *P. dilatata* (physical presence and chemistry) on recruitment of sympatric benthic organisms; (2) the allelopathic effects of its chemicals on competitors; and (3) chemotactic responses of the non-indigenous brittle star, *Ophiothela mirabilis*. Early establishment of benthic species was influenced on substrates around live *P. dilatata* colonies and some effects could be attributed to the gorgonian’s secondary metabolites*.*In addition, the gorgonian chemicals also exerted an allelopathic effect on the sympatric zoanthid *Palythoa caribaeorum,* and positive chemotaxis upon *O. mirabilis*. These results indicate multiple ecological roles of a chemically-defended gorgonian on settlement, sympatric competitors, and non-indigenous species.

## Introduction

Gorgonians (octocoral sea fans) are benthic marine invertebrates that occur in a wide range of habitats, from Arctic (Andrews et al., 2002) to Antarctic regions (Elias-Piera et al., 2013), and from intertidal waters (Yoshioka, 2009) down to deep-sea abysses (Mortensen & Buhl-Mortensen, 2005). However, most gorgonian species occur in tropical and sub-tropical marine waters (Alderslade, 1984). Given their diverse ecological range, many factors have been reported as being involved in the distribution of gorgonians, such as irradiance (Lasker, 2003), depth (Mortensen & Buhl-Mortensen, 2005), temperature (Ferrier-Pagés et al., 2009), substrate availability (Weinbauer & Velimirov, 1996), bottom sediment transport (Yoshioka, 2009) and topography (Tong et al., 2012). However, the ecological success of gorgonians has also been attributed to their evolutionary or adaptive production of highly diversified secondary metabolites, which exhibit multiple ecological roles (Coll, 1992).

Gorgonians are known as a chemically prolific group because they possess a wide array of compounds, primarily composed of lipids, steroids and terpenes (Blunt et al., 2015). That several species of gorgonians produce fish deterrents has been known for over 30 years (Gerhart, 1984). Defensive chemicals have since been identified in gorgonian species distributed worldwide, including in the Caribbean (e.g., Pawlik, Burch & Fenical, 1987, Pacific (e.g., Bright-Diaz, Strychar & Shirley, 2011), and the western North (Gerhart & Coll, 1993) and South Atlantic (Maia, 2001) regions. However, several specialist consumers of gorgonians are not affected by these chemicals, such as the gastropod, *Cyphoma gibbosum* (Harvell & Suchanek, 1987), the butterflyfish, *Chaetodon capistratus* (Lasker, 1985), the nudibranch, *Tritonia hamnerorum* (Cronin et al., 1995), and the bristleworm, *Hermodice carunculata* (Vreeland & Lasker, 1989). Such predators tend to inflict small-scale, localized damage on gorgonian colonies. Furthermore, a specialized dendronotid nudibranch, *T. hamnerorum*, which occurs in high densities on the sea fan *Gorgonia ventalina,* is able to store a sesquiterpene from this host in its tissues that effectively deters the common predatory reef fish *Thalassoma bifasciatum* (Cronin et al., 1995).

In addition to antipredator defenses, gorgonian chemicals (extracts and metabolites) are known to play a role as antifouling agents. For example, crude extracts of several Caribbean gorgonians exhibit varied antimicrobial activities (e.g.,  Aceret, Sammarco & Coll, 1995; Jensen et al., 1996), while pregnene glycosides from the gorgonian *Mu ricea fruticosa* inhibit the growth of the marine diatom, *Phaeodactylum tricornutum*, which is a potential fouling organism (Bandurraga & Fenical, 1985). Antifouling properties have also been attributed to *Gorgonia* spp. crude extracts due to growth inhibition of a pathogenic fungus, *Aspergillus sydowii* (Kim et al., 2000), with similar effects shown for extracts from *Echinogorgia* sp., *Ctenocella* cf. *umbraculum,* and *Subergogia suberosa* on various fungi (Koh et al., 2002). Some terpenoids present in octocorals are also known as allelochemicals, which promote tissue necrosis upon contact with neighboring scleractinians or even in the absence of contact, through the water column (Sammarco & Coll, 1992).

From gene to community level, the secondary metabolites produced by marine organisms are important phenotypic expressions that lead to insights about the forces that drive ecological systems and the evolution of marine environments. These distinct levels of organization have been explored separately in marine chemical and ecological studies. However, it is possible that gorgonian chemicals have important and cascading effects in the marine environment.

The gorgonian *Phyllogorgia dilatata* (Esper, 1806) is endemic to the southwestern Atlantic and its long-lasting beds frequently amass such a high density of colonies that a canopy forms on subtropical rocky reefs of Arraial do Cabo, northern Rio de Janeiro state (Cassola et al., 2016). Previous studies have indicated that chemicals from this sea fan exhibit defensive properties against fishes and fouling (Pereira et al., 2002; Epifanio et al., 1999; Epifanio, Da Gama & Pereira , 2006), therefore a multiple defensive role of these metabolites might be expected (Engel & Pawlik, 2000).

Although *P. dilatata* aggregations undoubtedly transform the reef landscape, little has been done to understand their influence on the understory community. We hypothesized that chemicals from *P. dilatata* exert an influence at the community level by: (1) influencing the recruitment of benthic marine organisms in its vicinity; (2) allelopathic effects on the growth of potential competitors; and (3) eliciting a chemotactic response on the associated brittle star, *Ophiothela mirabilis*.

## Materials and methods

### Study site

**Figure 1:**
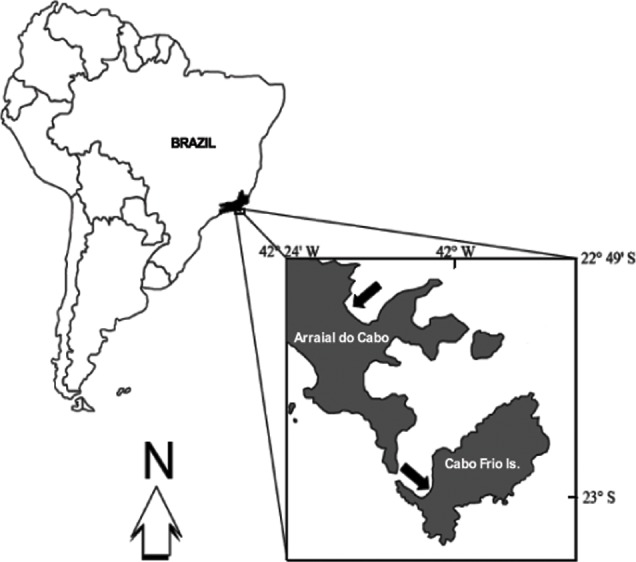
Study sites. Upper arrow indicates the Prainha site. Lower arrow indicates IEAPM field laboratory.

All fieldwork was carried out at Arraial do Cabo (22°58′35″S, 42°00′00″W), Rio de Janeiro, Brazil (Fig. 1). This area is well known for the occurrence of coastal upwelling and, therefore, harbors distinct reef assemblages. Some sites are currently protected under the status of an Extractivist Marine Reserve, but the local ecosystem is still threatened by illegal fishing and commercial ports.

Collection and manipulative experiments were performed on moored experimental structures within shallow rocky reefs (2–6 m depth) among *P. dilatata* aggregations at Prainha Beach and also at the field laboratory of Instituto de Estudos do Mar Almirante Paulo Moreira (IEAPM). All field activities were performed between October 2008 and December 2009.

### Extraction

Two large colonies of *P. dilatata* were randomly collected from within 2–4 m depth at Prainha Beach (A. do Cabo), by free diving. An aliquot of 765 ml of *P. dilatata* fresh tissue (excluding basal, attachment parts) was separated for extraction using a graduated cylinder filled with distilled water. After the crude extract was obtained (see below), it was used to reconstitute natural volumetric levels of chemical contents in experimental gels (Henrikson & Pawlik, 1995). The biological material was then allowed to dry in the dark at room temperature to avoid photolysis and thermal degradation of the secondary metabolites. Dried material was then submitted to exhaustive and successive extractions using the organic solvents hexane, dichloromethane (DCM), and methanol (MeOH), separately, to extract chemicals from a wide polarity spectrum. The solvents were later eliminated in a rotatory evaporator under reduced pressure, and the extracts were weighed and combined (referred to as ‘crude extract’ hereafter).

### Allelopathy experiments

**Figure 2:**
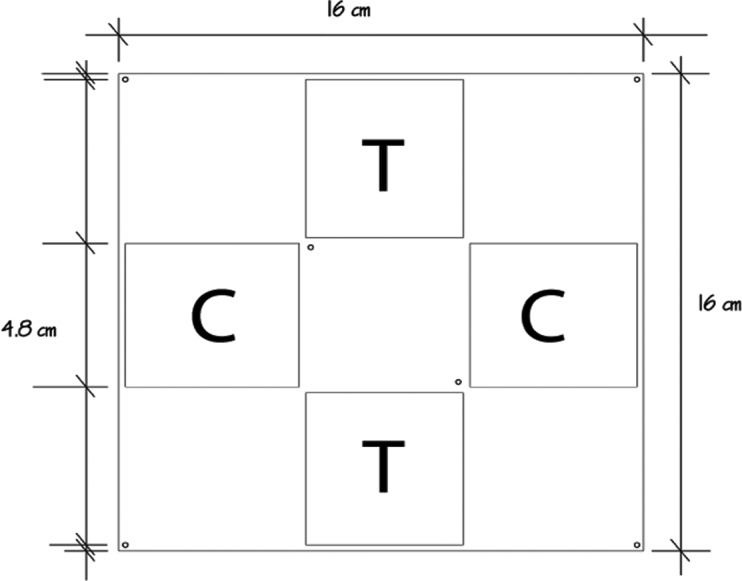
Allelopathy test plate bearing four diffusion chambers. *Palythoa caribaeorum* was fixed at the center. Treatment (T) and control (C) were chambers with and without crude extract of *Phyllogorgia dilatada*, respectively.

**Figure 3:**
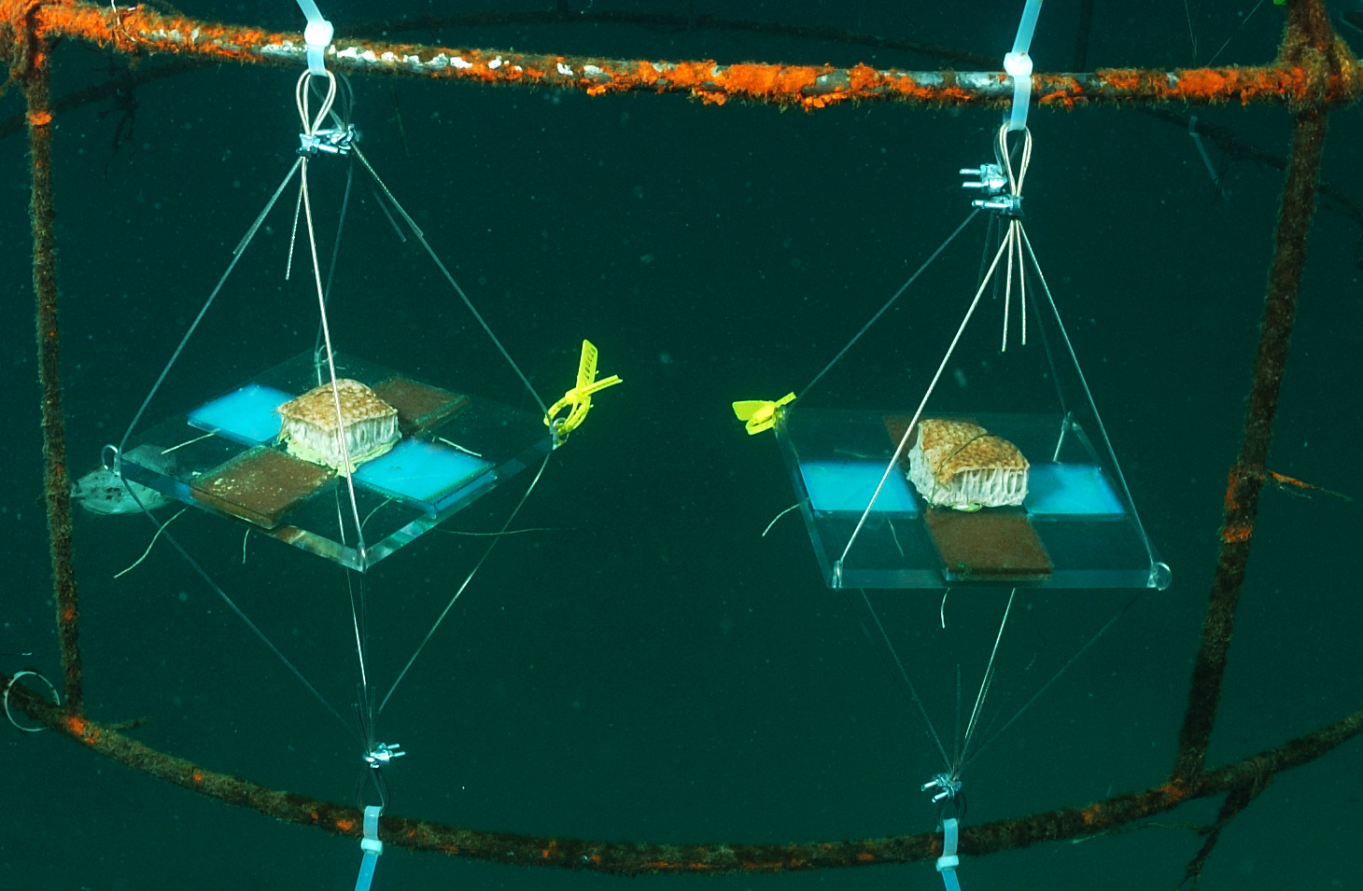
Allelopathy test plates on anchored structures at the IEAPM field laboratory.

The allelopathic effect of the crude extract of *P. dilatata* was evaluated on the sympatric zoanthid *Palythoa caribaeorum*. We used square acrylic panels measuring 16 cm and containing four equidistant diffusion chambers (4.8 × 4.8 × 0.8 cm) separated by a central plate (Fig. 2), a method previously designed to test allelopathy in sponges (Engel & Pawlik, 2000). A square fragment of *P. caribaeorum* was attached on the central plate. Two opposing wells (diffusion chambers) were filled with extract (treatment) gels while the remaining two were used for control gels. Natural volumetric concentrations of crude extract from *P. dilatata* were incorporated into a phytagel™ (Sigma Chemical Co.), prepared by mixing 1.5 g of this gel with 80 ml of distilled water to obtain the treatment. Control gels were prepared similarly, but without the addition of extract. On each plate, the competitor *P. caribaeorum* was allowed to overgrow paired extract-treated and control gels from the central plate. The experiment took place at the IEAPM field laboratory, where 10 plates were attached to moored structures (Fig. 3). Photographs were taken with SCUBA over the course of two months, using a Nikon D-70 digital camera with 16 mm lenses, to monitor colony growth of *P. caribaeorum* on chambers. To prevent distortion effects for photography, the focal distance was standardized to 25 cm. The plates were placed in the center of the field of view, as calibrated in preview trials. The response variable used to characterize the allelopathic effect of *P. dilatata* on *P. caribaeorum* was the growth of this zoanthid as total area of tissue on control chambers compared to those treated with crude extract. Growth was estimated by measuring the total area of *P. caribaeorum* cover over the diffusion chamber with *Image-J* software. A scale calibration was performed within each chamber according to the known dimensions to prevent distortion effects. Nine cups containing crude extract gels with the same volume of the plate chambers as described above were fixed on experimental structures as diffusion controls (see Da Gama et al., 2002), to ensure that allelochemicals from crude extract were preserved in gels throughout the whole experiment. The cups were retrieved from the field, the contents re-extracted and analyzed by Thin Layer Chromatography (TLC). After being transferred from the reef to the 10 experimental plates, we allowed 16 days for colonies to build coenosarc edges and fully heal the transplantation wounds before beginning photographic monitoring. Only six replicates fully recovered and were monitored further.

A non-parametric Wilcoxon signed-rank test was used to assess differences in growth between treatment and control chambers.

### Recruitment experiments

#### Live colony

**Figure 4:**
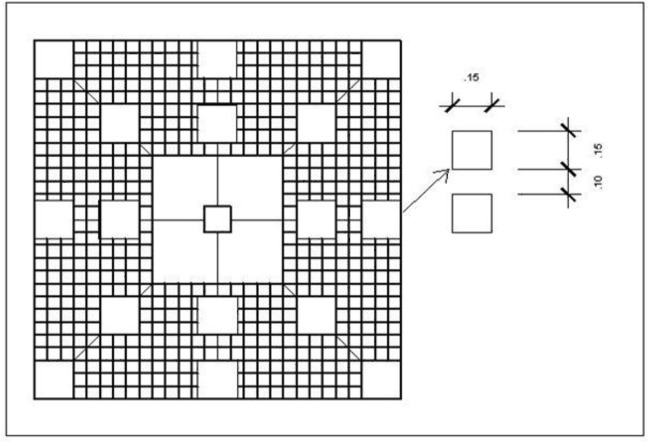
Experiment design for recruitment test near live colonies. Steel mesh structures with plastic panels were installed around a *Phyllogorgia dilatata* live colony or rubber model, on a steel mesh frame anchored to the substrate.

Two colonies of live *P. dilatata* (ca. 50 cm high and 40 cm wide) were randomly selected. A 1.3 × 1.3 m steel mesh was placed around both and anchored to the substrate. To investigate the possible effects of this gorgonian on the establishment of benthic organisms,., 16 PVC panels (15 × 15 cm) were installed over the mesh at 5 and 30 cm from the colony, an arrangement similar to that devised by Maida, Sammarco & Coll (1995) to assess allelopathic effects of soft corals on recruitment of scleractinian corals (Fig. 4).

As a control for the gorgonian’s tridimensional structure effect, two rubber mimics of the sea fan were designed, cut, and anchored to the substrate. Several materials were investigated until we found one (ethylene-vinyl acetate rubber) that behaved similarly to the waving motion of live gorgonians underwater in response to prevalent currents. In addition, the mimics matched the color of live gorgonians. Around each rubber mimic, another set of 1.3 m-steel mesh and 16 panels (as for the live *P. dilatata* assays) was prepared secured. Both treatment and control structures were installed by SCUBA diving, at approximately 6 m depth, among *P. dilatata* aggregations at Prainha Beach, Arraial do Cabo.

Panels were sampled for percent cover of settled taxa after 145 days by visual estimation. We excluded 1 cm of the edges from the sampling to avoid any edge effects on settling communities (Fagan, Cantrell & Cosner, 1999). Percent cover values were attributed to each taxon in relation to total panel area. For rare taxa, we assigned a minimal value of 1% only to record their presence on the sampling surfaces.

#### Crude extract

**Figure 5:**
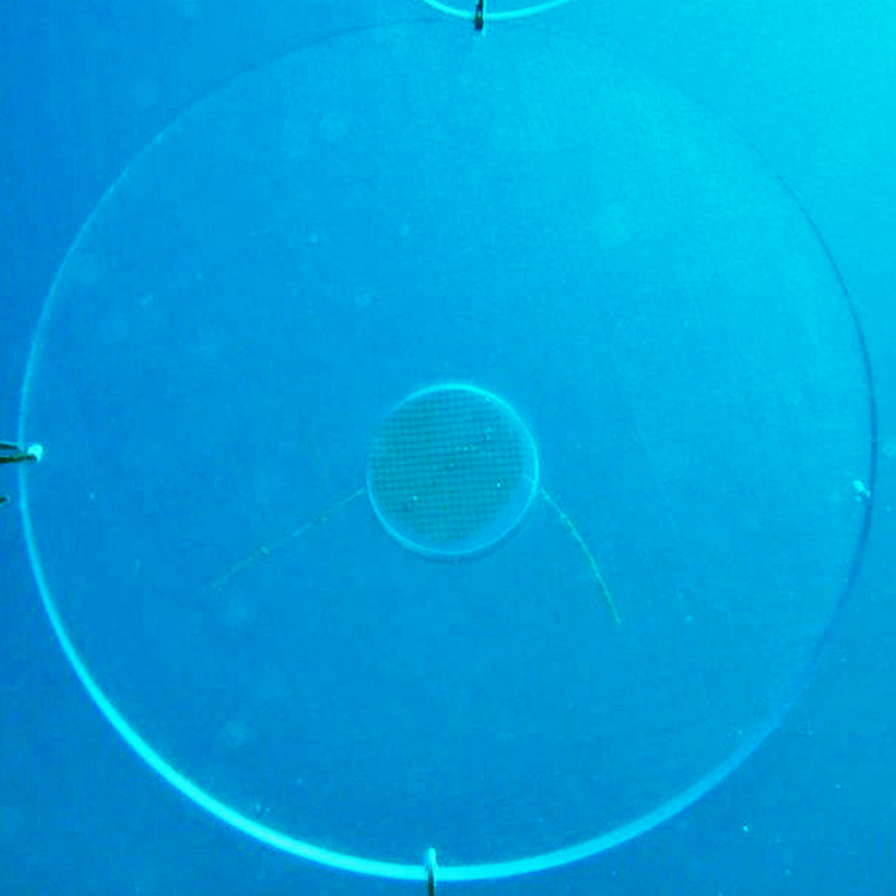
Disk-shaped acrylic panel with a coaxial diffusion chamber containing phytagel™ designed for the recruitment experiment.

We designed an experimental unit that consisted of an acrylic disk bearing a round diffusion chamber in the center (*viz*. (Engel & Pawlik, 2000) to specifically investigate the influence of the gorgonian crude extract on recruitment of other benthic organisms. The disks were colorless and made of a hard, laser-cut and inert material to ensure the replicates were identical in size, appearance and texture and to avoid any interference with settlement cues other than the sea fan extract. Disks were 25 cm in diameter and 1 cm thick, and the diffusion chambers were 5 cm in diameter and coaxial with the acrylic disk (Fig. 5).

**Figure 6:**
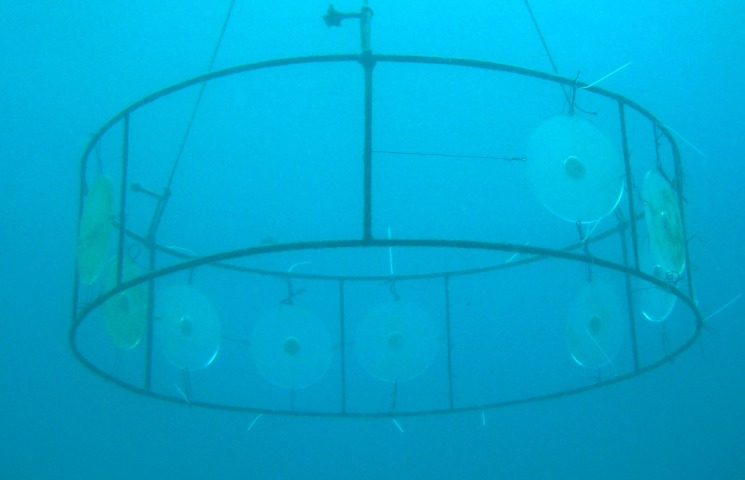
Acrylic disks on anchored structures at the IEAPM field laboratory.

Natural volumetric concentrations of *P. dilatata* crude extract were incorporated into phytagel™. The phytagel was prepared by adding and mixing 1.5 g of this gel to 35 ml of distilled water, and then poured inside the disk diffusion chambers. Only one side of the gel was exposed to currents so the diffusion rates of the crude extract into the water column were reduced, thereby simulating natural conditions of diffusion from living marine organisms (e.g., Da Gama et al., 2002). Control gels were prepared in the same way, but without the addition of extract. Experiments took place at the IEAPM field laboratory, where the 12 disks (6 disks per treatment) were randomly attached to an underwater structure at 5 m depth (Fig. 6).

After 45 days, the disks were retrieved and immediately transported to the lab to survey settled organisms. The 1-cm border was excluded to avoid edge effects. Each disk was visually inspected under a stereomicroscope to visually estimate percent cover of each taxon.

#### Statistical analyses

Data from both experiments (settlement panels with live colonies and acrylic disks with crude extracts) were analyzed in the same manner, focused in exploratory data analyses. In order to elucidate general differences between benthic assemblages in the vicinity of live colonies and those near mimics (or between extract-treated or control disks), data were plotted as Nonmetric Multidimensional Scaling (nMDS) graphics. Data were square-root or fourth-root transformed prior to analysis, and the plots are depicted according to a Bray Curtis similarity matrix. Percent values for representative sample groups were obtained using SIMPER analysis in PRIMER software v.6.1.6. Data from all 64 panels of the live colony experiment were analyzed, with 32 panels as treatments of *P. dilatata* presence and the remaining 32 as gorgonian mimics (controls). The crude extract experiment consisted of 10 disks treated with *P. dilatata* extract, with the same number of controls.

As SIMPER analysis indicated the taxa that contributed most to the differences between treatments, we performed an ANOVA with the percent cover of that taxon alone in both experiments. Data were logit-transformed prior to analyses in order to meet assumptions of normality and variance homogeneity (assessed via Shapiro–Wilk test and the Cochran *C* and Levene tests, respectively).

### Chemotaxis experiment

The invasive brittle star *Ophiothela mirabilis* is bright yellow/orange and inhabits octocoral branches in broad daylight. Individuals (oral disk diameter ca. 2 mm, arms length up to 10 mm) were collected from *P. dilatata* colonies and transported to the laboratory inside plastic containers with local seawater. To assess the existence of chemotactic responses of this brittle star to the gorgonian chemicals, the crude extract was incorporated into 1-cm ^3^ cubes of phytagel™ (refer to the allelopathy procedure described above), and placed in a Petri-dish paired with an untreated phytagel™ cube (control) on the opposite side. Dishes were filled with seawater.

Each *O. mirabilis* individual was positioned at the center of a 12-cm diameter Petri dish, between control and treatment choices and the behavior of the brittle stars was monitored. The movement of *O. mirabilis* from the center of the arena towards a preferred side (with extract or control) was observed within 2 mins after the specimen was placed into the arena and measured as the proportion of individuals that moved to either side. The test was repeated with 40 different individuals of *O. mirabilis*, and each individual was tested once only. Dishes were rinsed with seawater and placement of new gels (extract and control) was alternated to avoid any bias. The resulting proportion was analyzed with a corrected chi-square test (χ^2^) comparing observed data with expected frequencies under the null hypothesis of equal response of *O. mirabilis* to treated and control gels.

## Results

### Allelopathy experiments

**Figure 7:**
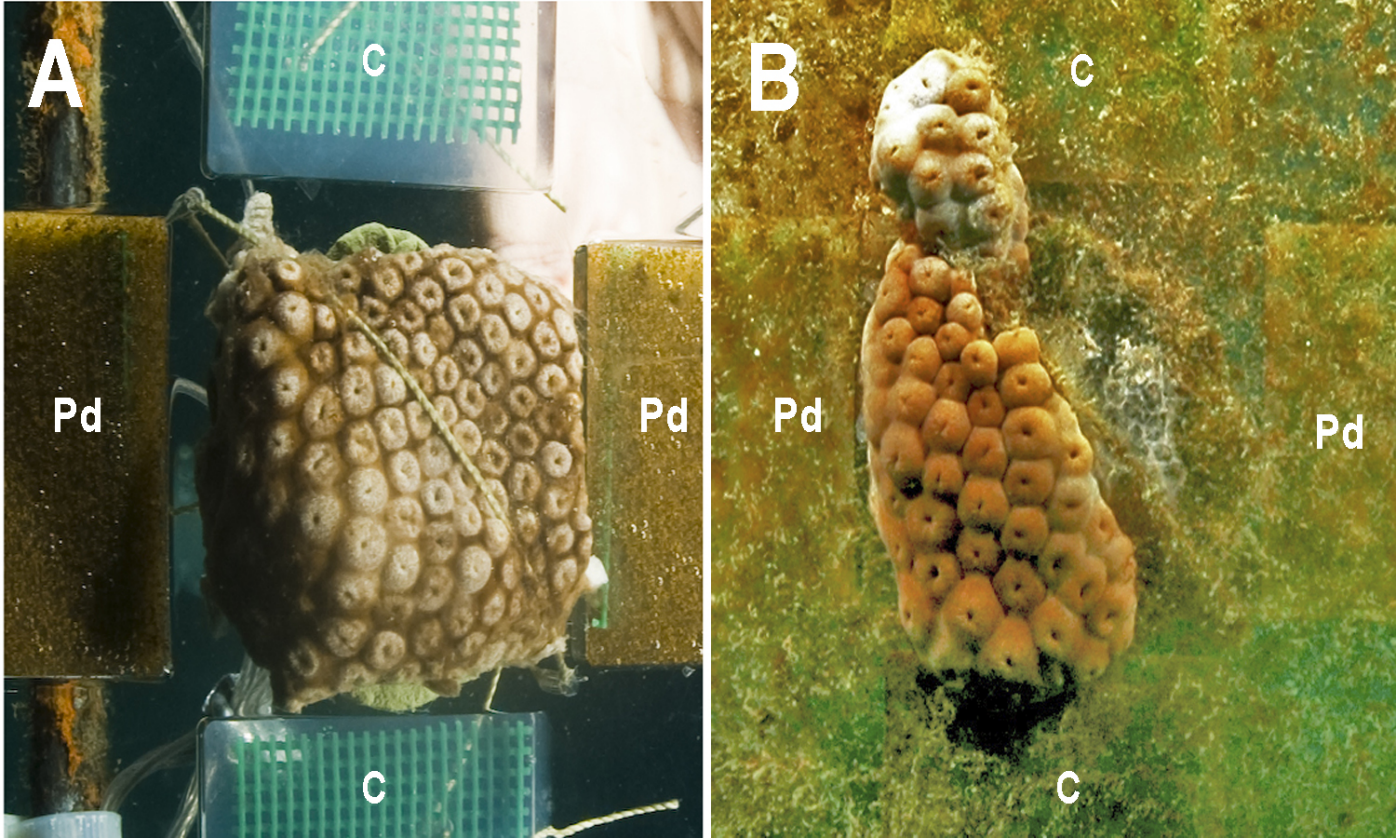
The zoanthid *Palythoa caribaeorum* fixed to an experimental unit at the beginning of the experiment (T0, A), and after 42 days (T2, B). (Pd) *Phyllogorgia dilatata* crude extract; (C) untreated gels.

**Figure 8:**
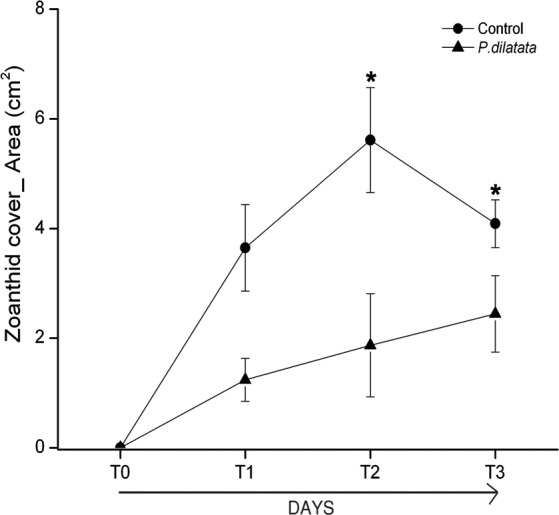
Total area of zoanthid *P. caribaeorum* tissue coverage over diffusion chambers treated with *P. dilatata* crude extract (triangles) and over controls (circles). (∗) = Wilcoxon test, zoanthid cover was higher for extract-treated chambers compared to controls at 42 days (T2) (*Z* = 2.2, *p* = 0.03, *n* = 6) and 57 days (T3) (*Z* = 2.2, *p* = 0.03, *n* = 6). Error bars = S.E.

At the first monitoring (T1, 16 days), there was no difference between cover of the zoanthid *P. caribaeorum* over diffusion chambers with and without crude extract of *P. dilatata* (Wilcoxon test: *Z* = 1.8, *p* = 0.074, *n* = 6). After 42 days (T2), coverage of *P. caribaeorum* on control chambers was significantly higher than for chambers with the crude extract of *P. dilatata* (Wilcoxon test: *Z* = 2.2, *p* < 0.05, *n* = 6) (Fig. 7). Although *P. caribaeorum* cover declined over the control chamber thereafter, cover remained significantly higher compared to the extract-treated chamber at T3 (57 days; Wilcoxon test: *Z* = 2.2, *p* < 0.05, *n* = 6) and zoanthid colonies were still healthy. The total area of *P. caribaeorum* tissue over chambers at each monitoring event is given in Fig. 8. Diffusion control gels retrieved from the field were re-extracted and thin layer chromatography (TLC) analysis revealed that overall chemical profiles were similar throughout the experiment. Although percentage mean retention was not calculated, the same procedure used in Da Gama et al. (2002) was categorically repeated in order to increase compounds preservation in gels. These authors retrieved an average of 43% of original extracts after 6 weeks. According to Henrikson & Pawlik (1995), phytagel is a resilient material, also standing periods of up to 6 weeks (42 days) with no evidence of degradation.

### Recruitment experiments

#### Live colonies

**Figure 9:**
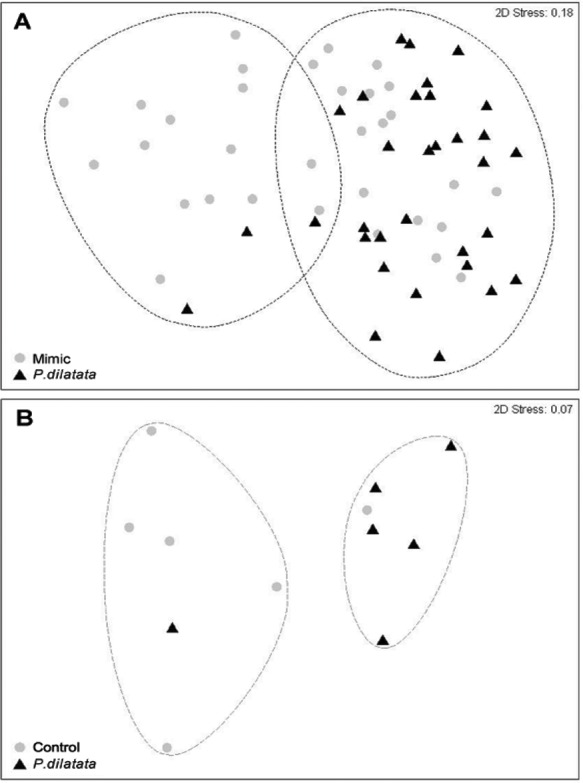
(A) Fouling community over recruitment panels near live colonies of *P. dilatata* and mimics. Samples are grouped within 60% similarity contours. Data are square-root transformed. (B) Fouling community over disks treated with crude extract and controls. Samples are grouped within 75% similarity contours. Data are fourth-root transformed.

After 145 days, the total cover of seaweeds was consistently higher near to live colonies of *P. dilatata* (>70%), while those near sea fan mimics had similar percentages of cover (ca. 50%) of algae and invertebrate taxa (Table 1). Two distinct groups within 60% similarity were identified from the non-metric multidimensional ordination (nMDS) plot (Fig. 9A). Most organisms that settled near mimics clustered together, and the same pattern was apparent for those near live colonies, with few exceptions. The two red seaweeds, *Spyridia* sp. and *Centroceras clavulatum*, and the Serpulidae polychaetes accounted for the main differences between communities settled on treatment and control panels (ANOSIM R = 0.181, *p* < 0.005). According to the SIMPER analysis of species contributions, *Spyridia* sp. contributed more than 26% to the average similarity (65.98%) among settled organisms near live gorgonoid colonies. Tube dwelling Serpulidae, which were more abundant on panels near mimics than near *P. dilatata* (ANOVA, *df* = 1; *F* = 21.07; *p* < 0.005), also contributed with 11.65% to the average dissimilarity (35.83%) between groups.

**Table 1:** Mean percent cover (%) with standard deviation (± SD) for taxa across treatments and controls of recruitment experiments on PVC panels and acrylic disks evaluating the effect of *Phyllogorgia dilatata* on settlement. Percent cover values for empty space/biofilm not assessed. Total Algal and Invertebrate cover values as sum of percent cover of all taxa within treatment. (−) absence of taxa.

Taxa	PVC panels Live colonies (Mean % cover ± SD)	PVC panels Mimics (Mean % cover ± SD)	Acrylic disks^a^ Crude extract (Mean % cover ± SD)	Acrylic disks Control (Mean % cover ± SD)
Algae				
*Ulva intestinalis*	0.09 ± 0.5	–	–	–
*Ulva* sp.	1.38 ± 1.4	2.69 ± 2.0	–	–
*Centroceras clavulatum*	8.13 ± 9.8	4.41 ± 7.2	–	–
*Cladophora* sp.	2.38 ± 2.0	2.34 ± 2.0	1.9 ± 1.0	1.3 ± 0.6
Ectocarpaceae	0.03 ± 0.2	–	28.4 ± 10.7	18.1 ± 9.1
Corallinaceae (CCA)	1.56 ± 2.0	2.22 ± 1.7	1.3 ± 1.2	0.7 ± 1.3
*Ceramium sp.*	–	–	1.1 ± 1.0	0.3 ± 0.3
*Hypnea cervicornis*	1.53 ± 2.2	2.22 ± 2.6	–	–
*Jania adhaerens*	1.50 ± 2.4	0.44 ± 0.7	–	–
*Laurencia obtusa*	0.69 ± 1.3	2.06 ± 3.0	–	–
*Spyridia* sp.	32.97 ± 16.0	20.19 ± 11.0	1.0 ± 0.6	0.6 ± 0.7
*Padina gymnospora*	0.50 ± 0.9	0.44 ± 0.8	–	–
*Sargassum* sp.	0.97 ± 1.2	1.13 ± 1.0	–	–
*Colpomenia sinuosa*	0.03 ± 0.2	0.03 ± 0.2	0.1 ± 0.1	–
*Dictyota* sp.	1.06 ± 2.0	0.59 ± 1.1	–	–
*Diatomeae*	–	–	23.6 ± 9.6	15.1 ± 9.5
*Cyanobacteria*	–	–	0.7 ± 0.7	0.4 ± 1.0
Crustacea				
*Amphibalanus* sp.	0.16 ± 0.6	0.66 ± 1.4	–	–
*Balanus trigonus*	2.75 ± 4.1	2.56 ± 2.8	2.5 ± 1.7	0.7 ± 0.9
Tunicata				
Ascidiacea	0.03 ± 0.2	–	0.1 ± 0.2	–
Bryozoa				
*Membranipora membranacea*	–	–	0.2 ± 0.4	–
*Schizoporella errata*	0.31 ± 1.8	–	–	–
Bryozoa 1	0.16 ± 0.6	0.06 ± 0.3	–	–
Hydrozoa				
*Obelia sp.*	–	–	33.7 ± 9.5	58.1 ± 18.7
Polychaeta				
Serpulidae	15.56 ± 8.8	32.81 ± 18.7	0.1 ± 0.1	0.1 ± 0.1
Total Invertebrate cover (%)	18,97	36,09	36,6	58,9
Total Algal cover (%)	52.81	38.75	52.76	39.81

**Notes.**

a In order to assess the radius of action of the gorgonian extract, we originally sampled the disk area arbitrarily subdivided in three 3-cm concentric rings around the central diffusion well. As initial analyses did not reveal differences among rings, data were pooled.

#### Crude extract

After 45 days, 14 taxa had settled on the disks, including algae, cyanobacteria, benthic diatoms, hydrozoans, polychaetes, bryozoans, barnacles, and ascidians (Table 1). Overall, settlement of algal species was higher on extract-treated disks than on controls (Table 1). The bryozoan *Membranipora membranacea,* an unidentified tunicate species and the brown alga *Colpomenia sinuosa* only settled on control disks.

**Figure 10:**
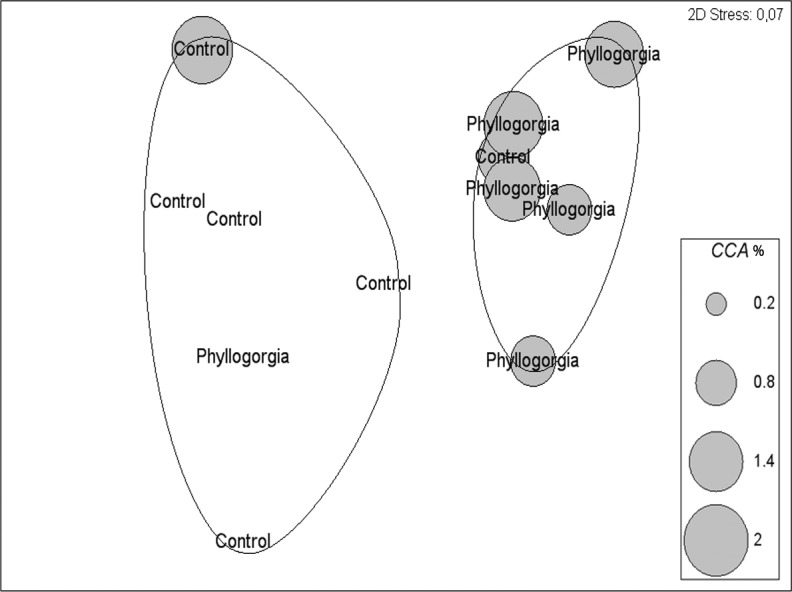
Percent cover of coralline crustose algae (CCA) over disks treated with crude extract (*Phyllogorgia*) and controls, within 75% similarity groups.

Two distinct groups within 75% percent similarity were apparent in the non-metric multidimensional ordination (nMDS) plot (Fig. 9B). However, the communities settled on treatment and control disks (*n* = 6 replicates per treatment) barely differed (ANOSIM R = 0.231, *p* = 0.056). According to the analysis of species individual contributions (SIMPER), settled communities on control disks had an average similarity of 78.4%, with *Obelia* sp. contributing more than 33% to that similarity. Similarity of settled communities near *P. dilatata* extracts was over 81%, with the hydrozoan *Obelia* sp. also contributing 21.74% to this pattern. The main differences between control and treatment could be attributed to barnacles and crustose coralline algae (CCA) that together accounted for over 25% of the dissimilarity observed between groups. CCA, cyanobacteria and *Balanus trigonus* recruited more on extract-treated disks (Table 1, Fig. 10).

#### Chemotaxis experiment

The invasive ophiuroid, *O. mirabilis*, exhibited a directional preference in response to exposure to *P. dilatata* crude extract. Only brittle stars that made a clear choice were used in data analysis (*N* = 40), of which 27 reacted positively towards the extract-treated cubes, crawling the entire intervening distance and touching the gels. Thirteen individuals moved toward the controls. A statistically significant higher proportion of brittle stars moved toward the extract (χ^2^ = 5.9, *df* = 1, *p* = 0.015).

## Discussion

Besides the possible effects that known *P. dilatata* substances may have upon the adjacent community, the very presence of this species must be taken into account due to its physical structure and the tridimensionality that *P. dilatata* beds add to subtropical rocky reefs in Brazil. Here, we hypothesized that *P. dilatata* colonies and their chemicals may exert an effect at the community level.

Most canopy-forming species exhibit some seasonality, allowing understory communities to develop within their sphere of influence until they are eventually washed out after a few months. These canopies provide microhabitats with distinct light and water movement regimes (Reed & Foster, 1984). Benthic communities associated with dense aggregations of canopy-forming species end up being shaped by such regimes in terms of taxa composition and abundance (Watt & Scrosati, 2013). In our study, we demonstrate that live colonies of *P. dilatata* do indeed affect recruitment, resulting in a slightly distinct understory benthic community.

Recruitment on panels near live colonies of *P. dilatata* at Prainha beach had a higher coverage of algal taxa compared to those near mimics, indicating that this sea fan can enhance the colonization of adjacent substrata by seaweeds. Territorial herbivorous fishes, such as *Stegastes fuscus* that is known to keep edible algal assemblages within a given perimeter, could benefit from higher seaweed colonization (Ceccarelli, Jones & McCook, 2005). Moreover, this newly-settled algae would provide food for several species of Pomacentridae, Scaridae, Kyphosidae and Acanthuridae occurring in the study area (Ferreira, Gonçalves & Coutinho, 2001), which also feed on algae (Ferreira & Gonçalves, 2006).

The majority of marine benthic species possess a life history that includes settlement and metamorphosis, which may occur in response to specific chemical or physical cues (Tebben et al., 2015; Whalan et al., 2015). A variety of chemical signals have been shown to induce marine invertebrate settlement, thus modulating or influencing the structure of benthic marine communities (Pereira, Da Gama & Sudatti, 2017). Our results are the first to report the influence of a gorgonian on settlement of benthic organisms on adjacent substrata.

The higher coverage of algae on extract-treated disks indicates a possible role of *P. dilatata* in attracting algal taxa to adjacent substrata. It has long been assumed that secondary metabolites produced by marine organisms may have a significant impact on the structure of benthic communities. Several laboratory studies have shown that larvae of benthic marine organisms use chemical cues to select settlement sites (e.g., Jouuchi, Satuito & Kitamura, 2007). Curiously, CCA settled more on extract-treated disks, contributing to a slight dissimilarity between the control and treatment groups. Further investigation is needed to confirm this finding, but it is possible that *P. dilatata* could stimulate substrate colonization by such pioneer species that provide habitat-specific cues and enhance settlement by reef builders (Tebben et al., 2015). On the other hand, Pereira et al. (2002) observed inhibition of CCA on gels with gorgonian extracts, while we found this taxon to be more abundant on panels near live gorgonians.

Calcareous tube-dwelling polychaetes (family Serpulidae) were significantly less abundant around *P. dilatata* than around its mimics. Pereira et al. (2002) also found an inhibitory effect of *P. dilatata* extracts on invertebrates (barnacles) in field experiments. It has been demonstrated that chemicals from soft corals inhibit the settlement of serpulids and algal propagules (Maida, Sammarco & Col, 2001). In contrast, higher algal recruitment by *Enteromorpha* and *Cladophora* was found for panels treated with terpenoids from the soft coral, *Sinularia flexibilis* (Michalek & Bowden, 1997). Notably, organic extracts from anthozoans may influence settlement, behavior, and growth of larvae and spores of several other sessile organisms, including conspecifics (Maida, Sammarco & Col, 2001; Fearon & Cameron, 1997). However, a diterpene isolated from *P. dilatata* (11 β, 12 β-epoxipukalide) has been shown to inhibit fouling (Epifanio, Da Gama & Pereira, 2006). Given that other unknown compounds in the crude extract positively influenced settlement in nearby substrata, it is possible that this compound plays a more specific role in keeping the gorgonian body devoid of overgrowth by epibionts or competitors upon close contact.

We demonstrated close contact inhibition through experiments with the zoanthid competitor, *Palythoa caribaeorum*. This anthozoan was chosen for this assay because it is a frequent and abundant component of benthic communities along the Brazilian coast (Pérez, Vila-Nova & Santos, 2005). Zoanthids build up large encrusting colonies that quickly overgrow several other benthic species, resulting in regime shifts in reef systems (Cruz et al., 2015). At Arraial do Cabo, *P. caribaeorum* overgrows organisms even on the lower mid-littoral zone (Mendonça-Neto & Da Gama , 2009). In addition, this species is chemically-defended and maintains high cover even under turbid environments (Bastidas & Bone, 1996).

Most alcyonarian sea fans frequently have an upright morphology that somewhat provides protection from other competitors. However, this strategy is not sufficient for diseased *P. dilatata* colonies, which are typically overgrown from base to top by encrusting species like CCA and milleporids (Cassola et al., 2016). It seems plausible that diseased gorgonians may reduce or even lose their chemical defense system, since the production costs are high (Pawlik, 1993).

Even though *P. dilatata* cannot completely inhibit competitors—a non-indigenous soft coral, *Chromonephthea braziliensis,* imposes chemically-mediated damage on *P. dilatata* tissue (Lages et al., 2006)—its chemical defenses inhibited growth of the native competitor *P. caribaeorum*. If inhibitory substances are present at the base of the colony where it attaches to the substrate, it is possible that in natural encounters *P. caribaeorum* can direct growth so its tissue is beyond the influence of the gorgonian’s allelochemicals. Engel & Pawlik (2000), using the same method, found that crude extracts from the sponge *Aplysilla longispina* inhibit growth of other conspecifics and tunicates. This might partly explain the co-existence of encrusting species in marine communities.

An additional ecological role of *P. dilatata* chemicals is the positive chemotactic response of the non-indigenous brittle star *O. mirabilis*. In general, the success of an introduced species is typically associated with its rates of reproduction, growth, mortality, and successful competition for resources with native species, as well as with the physical characteristics of the environment (Sammarco et al., 2015). However, habitat availability may also be important in predicting patterns amongst native communities and invasive species at biogeographic scales (Gribben, Simpson & Wright, 2015). This species of brittle star densely colonizes gorgonians and sponges on reefs of the Indo-West Central Pacific and Tropical Eastern Pacific, with non-indigenous populations also being reported for the Atlantic Ocean, from Caribbean to Brazilian shores (Hendler et al., 2012). Here, we provide evidence that chemical signals from an endemic benthic species, *P. dilatata*, might provide specific cues for *O. mirabilis* in pursuit of suitable habitat, thereby indirectly facilitating the success of this invasive species.

Although it has been suggested that brittle stars can build up long-lasting species-specific interactions with host octocorals (Mosher & Watling, 2009), this is probably not the case for *O. mirabilis* in Brazil because several benthic species from distinct taxonomic groups are known to host this invader (Mantelatto et al., 2016) . However, by being attracted to and living in association with a species chemically-defended against consumers, this brittle star benefits from *P. dilatata* through reduced predation, which facilitates its establishment in a new habitat.

Previous studies have shown that other octocoral species can increase the diversity of the fouling community (Maida, Sammarco & Col, 2001). Also, it has been suggested that chemically-defended species provide a refuge within which species vulnerable to predation can develop, protected from consumers (Littler, Taylor & Littler, 1986). According to our results, *P. dilatata* mainly influences the initial stages of succession near live colonies and one possible outcome is a richer algal assemblage. However, rather than protecting algae from local herbivores, *P. dilatata* attracts consumers to the understory community and thus bolsters reef biodiversity.

The endemic gorgonian *P. dilatata* might contribute to the maintenance of a stable community as a consequence of its perennial canopies (Knowlton, 1992). Additional manipulative experiments are required to fully understand the influence of *P. dilatata* over the surrounding community and to isolate the effects of physical structure, chemistry and biology.

##  Supplemental Information

10.7717/peerj.3186/supp-1 Supplemental Information 1 Image of *Phyllogorgia dilatata* in natural environment at Arraial do Cabo, RJ, Brazil

10.7717/peerj.3186/supp-2 Supplemental Information 2 Raw data of recruitment experiments and alleopathy Table 1. Live colonies recruitment experiment. Percent cover of each taxon per replicate for control plates (near mimics).
Table 2. Live colonies recruitment experiment. Percent cover of each taxon per replicate for treatment plates (near Phyllogorgia dilatata colonies)
Table 3. Crude extract recruitment experiment. Percent cover per replicate for acrylic disks.
Table 4. Total area cover (cm ^2^) of *Palythoa caribaeorum* over difusion chambers. Allelopathy experiment.

10.7717/peerj.3186/supp-3 Supplemental Information 3 Raw data of all experiments Table 1. Live colonies recruitment experiment. Percent cover per replicate for control plates near mimics.
Table 2. Live colonies recruitment experiment. Percent cover per replicate for treatment plates near live gorgonians.
Table 3. Crude extract recruitment experiment. Percent cover per replicate for acrylic disks.
Table 4. Total area cover (cm^2^) of *Palythoa caribaeorum* on wells over time.

## Additional Information and Declarations

### Field Study Permissions

The following information was supplied relating to field study approvals (i.e., approving body and any reference numbers): All fieldwork was carried out at Arraial do Cabo (22°58′35″ S, 42°95 00′00″W), Rio de Janeiro, Brazil (SISBIO 34321-1).

### Data Availability

The following information was supplied regarding data availability: The raw data has been supplied as Supplementary Files.
