# Supplementary figures and images for: Structuring effects of chemicals from the sea fan Phyllogorgia dilatata on benthic communities
Source: PeerJ. 2017 Apr 4;5:e3186. doi: 10.7717/peerj.3186 (PMC5382925; doi:10.7717/peerj.3186)

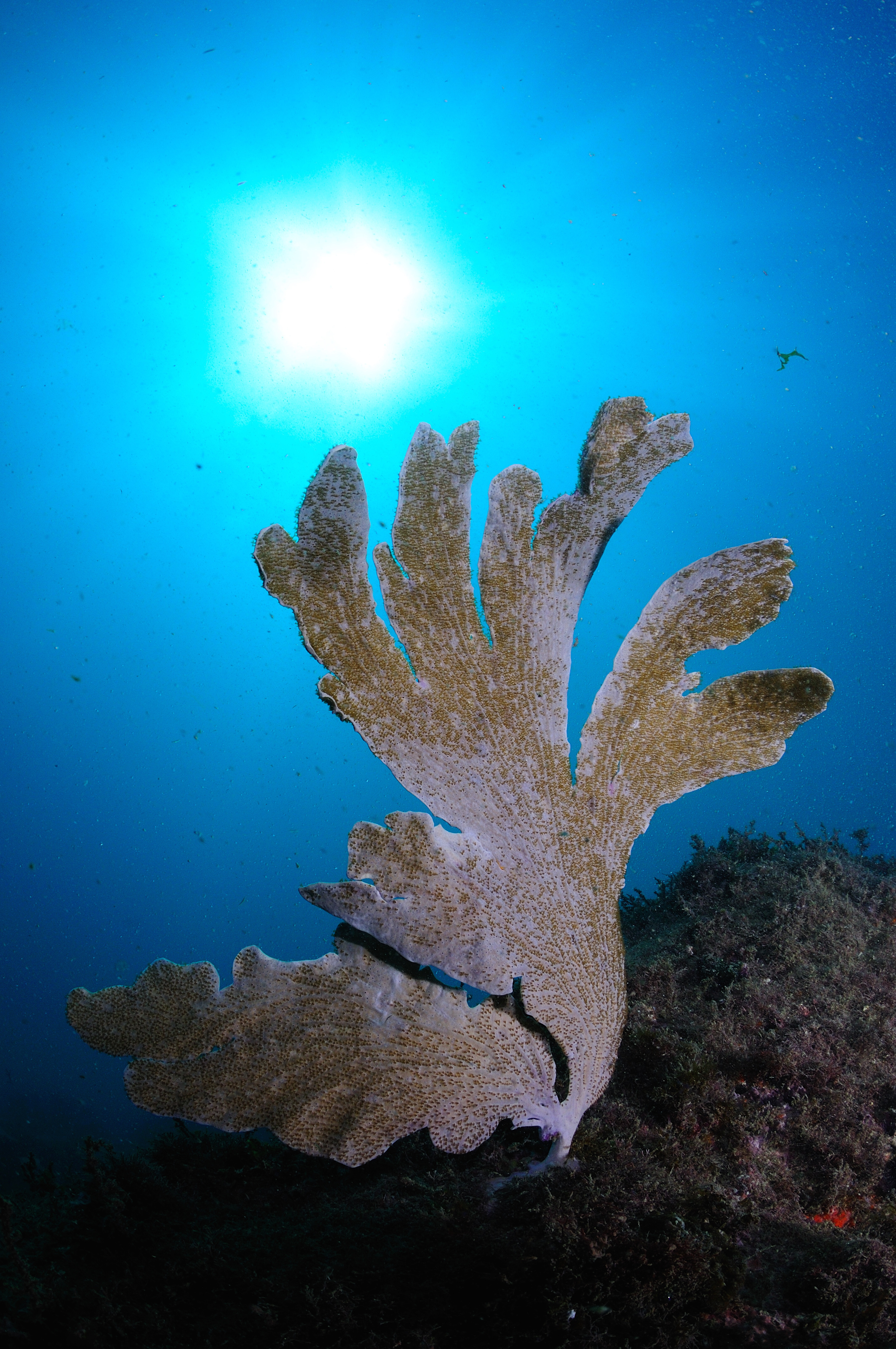

Supplement: Supplemental Information 1 [file peerj-05-3186-s001.jpg]
