# Supplementary material for: Structuring effects of chemicals from the sea fan Phyllogorgia dilatata on benthic communities
Source: PeerJ. 2017 Apr 4;5:e3186. doi: 10.7717/peerj.3186 (PMC5382925; doi:10.7717/peerj.3186)
Supplement: Supplemental Information 2 — Table 1. Live colonies recruitment experiment. Percent cover of each taxon per replicate for control plates (near mimics). Table 2. Live colonies recruitment experiment. Percent cover of each taxon per replicate for treatment plates (near Phyllogorgia dilatata colonies) Table 3. Crude extract recruitment experiment. Percent cover per replicate for acrylic disks. Table 4. Total area cover (cm 2) of Palythoa caribaeorum over difusion chambers. Allelopathy experiment. [file peerj-05-3186-s002.docx]

Table 1. Live colonies recruitment experiment. Percent cover per replicate for control plates.

|  | 1A | 1B | 1C | 1D | 1E | 1F | 1G | 1H | 1I | 1J | 1L | 1M | 1N | 1O | 1P | 1Q | 3A | 3B | 3C | 3D | 3E | 3F | 3G | 3H | 3I | 3J | 3L | 3M | 3N | 3O | 3P | 3Q |
| --- | --- | --- | --- | --- | --- | --- | --- | --- | --- | --- | --- | --- | --- | --- | --- | --- | --- | --- | --- | --- | --- | --- | --- | --- | --- | --- | --- | --- | --- | --- | --- | --- |
| *Spyridia sp.* | 5 | 15 | 10 | 5 | 35 | 25 | 45 | 20 | 25 | 15 | 25 | 20 | 20 | 20 | 35 | 20 | 1 | 20 | 10 | 0 | 45 | 20 | 15 | 20 | 25 | 20 | 15 | 5 | 35 | 30 | 25 | 20 |
| *Ulva intestinalis* | 0 | 0 | 0 | 0 | 0 | 0 | 0 | 0 | 0 | 0 | 0 | 0 | 0 | 0 | 0 | 0 | 0 | 0 | 0 | 0 | 0 | 0 | 0 | 0 | 0 | 0 | 0 | 0 | 0 | 0 | 0 | 0 |
| *Dictyota* sp. | 1 | 0 | 0 | 0 | 1 | 1 | 0 | 0 | 0 | 1 | 1 | 5 | 0 | 0 | 0 | 0 | 0 | 0 | 3 | 0 | 3 | 0 | 0 | 0 | 0 | 0 | 1 | 0 | 1 | 0 | 0 | 1 |
| *Centroceras clavulatum* | 0 | 5 | 3 | 0 | 3 | 10 | 1 | 10 | 10 | 10 | 15 | 5 | 1 | 0 | 1 | 15 | 0 | 1 | 0 | 0 | 0 | 0 | 30 | 20 | 0 | 0 | 0 | 1 | 0 | 0 | 0 | 0 |
| *Sargassum* sp. | 3 | 3 | 3 | 1 | 0 | 0 | 0 | 1 | 3 | 1 | 1 | 1 | 0 | 3 | 0 | 1 | 0 | 1 | 1 | 0 | 3 | 0 | 1 | 3 | 1 | 0 | 0 | 1 | 1 | 1 | 1 | 1 |
| *Hypnea cervicornis* | 3 | 5 | 3 | 1 | 1 | 3 | 1 | 3 | 3 | 1 | 5 | 5 | 1 | 0 | 3 | 10 | 1 | 0 | 1 | 1 | 0 | 0 | 10 | 5 | 0 | 1 | 1 | 1 | 1 | 0 | 1 | 0 |
| *Ulva* sp. | 3 | 3 | 10 | 5 | 0 | 0 | 0 | 1 | 1 | 3 | 1 | 1 | 3 | 1 | 3 | 5 | 5 | 3 | 1 | 3 | 1 | 1 | 3 | 1 | 5 | 3 | 3 | 3 | 3 | 3 | 5 | 3 |
| *Padina gymnospora* | 1 | 0 | 1 | 3 | 0 | 1 | 0 | 0 | 0 | 0 | 0 | 0 | 0 | 1 | 1 | 1 | 0 | 0 | 3 | 0 | 1 | 1 | 0 | 0 | 0 | 0 | 0 | 0 | 0 | 0 | 0 | 0 |
| *Laurencia obtusa* | 0 | 0 | 1 | 0 | 0 | 1 | 10 | 5 | 5 | 1 | 0 | 10 | 5 | 1 | 1 | 3 | 0 | 1 | 0 | 0 | 3 | 10 | 0 | 3 | 0 | 0 | 0 | 0 | 0 | 0 | 3 | 3 |
| *Ceramium sp.* | 0 | 0 | 0 | 0 | 0 | 0 | 0 | 0 | 0 | 0 | 0 | 0 | 0 | 0 | 0 | 0 | 0 | 0 | 0 | 0 | 0 | 0 | 0 | 0 | 0 | 0 | 0 | 0 | 0 | 0 | 0 | 0 |
| Serpulidae | 70 | 50 | 45 | 45 | 5 | 25 | 10 | 25 | 35 | 25 | 30 | 30 | 15 | 10 | 30 | 25 | 50 | 50 | 45 | 65 | 10 | 15 | 50 | 20 | 40 | 35 | 15 | 70 | 10 | 15 | 25 | 60 |
| *Amphibalanus* sp. | 1 | 1 | 5 | 0 | 1 | 0 | 0 | 0 | 1 | 1 | 3 | 0 | 0 | 5 | 0 | 0 | 0 | 0 | 0 | 0 | 0 | 0 | 3 | 0 | 0 | 0 | 0 | 0 | 0 | 0 | 0 | 0 |
| Biofilm | 10 | 5 | 10 | 0 | 65 | 30 | 40 | 30 | 40 | 45 | 20 | 35 | 0 | 60 | 15 | 15 | 0 | 3 | 3 | 15 | 75 | 90 | 30 | 20 | 35 | 15 | 0 | 5 | 60 | 60 | 45 | 3 |
| Corallinaceae (CCA) | 3 | 3 | 5 | 3 | 1 | 3 | 3 | 3 | 3 | 3 | 3 | 1 | 3 | 5 | 1 | 5 | 5 | 1 | 1 | 3 | 0 | 1 | 3 | 5 | 1 | 0 | 0 | 0 | 1 | 0 | 1 | 1 |
| *Cladophora* sp. | 0 | 5 | 5 | 1 | 1 | 3 | 1 | 0 | 0 | 1 | 1 | 1 | 3 | 3 | 1 | 10 | 3 | 3 | 3 | 3 | 3 | 3 | 3 | 3 | 1 | 3 | 3 | 1 | 3 | 3 | 0 | 1 |
| Ectocarpaceae | 0 | 0 | 0 | 0 | 0 | 0 | 0 | 0 | 0 | 0 | 0 | 0 | 0 | 0 | 0 | 0 | 0 | 0 | 0 | 0 | 0 | 0 | 0 | 0 | 0 | 0 | 0 | 0 | 0 | 0 | 0 | 0 |
| *Jania adhaerens* | 0 | 0 | 0 | 0 | 0 | 1 | 1 | 0 | 1 | 1 | 0 | 1 | 3 | 1 | 1 | 0 | 0 | 1 | 0 | 0 | 0 | 1 | 0 | 1 | 0 | 0 | 0 | 0 | 1 | 0 | 0 | 0 |
| *Balanus trigonus* | 3 | 3 | 3 | 10 | 1 | 1 | 5 | 1 | 0 | 3 | 1 | 0 | 5 | 0 | 3 | 1 | 1 | 0 | 5 | 5 | 0 | 0 | 3 | 0 | 0 | 5 | 5 | 10 | 0 | 5 | 0 | 3 |
| Bryozoa 1 | 0 | 0 | 0 | 1 | 0 | 0 | 0 | 0 | 0 | 0 | 0 | 0 | 0 | 0 | 0 | 0 | 0 | 0 | 0 | 0 | 0 | 0 | 0 | 0 | 0 | 1 | 0 | 0 | 0 | 0 | 0 | 0 |
| Ascidia | 0 | 0 | 0 | 0 | 0 | 0 | 0 | 0 | 0 | 0 | 0 | 0 | 0 | 0 | 0 | 0 | 0 | 0 | 0 | 0 | 0 | 0 | 0 | 0 | 0 | 0 | 0 | 0 | 0 | 0 | 0 | 0 |
| *Schizoporella errata* | 0 | 0 | 0 | 0 | 0 | 0 | 0 | 0 | 0 | 0 | 0 | 0 | 0 | 0 | 0 | 0 | 0 | 0 | 0 | 0 | 0 | 0 | 0 | 0 | 0 | 0 | 0 | 0 | 0 | 0 | 0 | 0 |
| *Colpomenia sinuosa* | 0 | 0 | 0 | 0 | 0 | 0 | 0 | 0 | 0 | 0 | 0 | 0 | 0 | 0 | 0 | 1 | 0 | 0 | 0 | 0 | 0 | 0 | 0 | 0 | 0 | 0 | 0 | 0 | 0 | 0 | 0 | 0 |

|  | 2A | 2B | 2C | 2D | 2E | 2F | 2G | 2H | 2I | 2J | 2L | 2M | 2N | 2O | 2P | 2Q | 4A | 4B | 4C | 4D | 4E | 4F | 4G | 4H | 4I | 4J | 4L | 4M | 4N | 4O | 4P | 4Q |
| --- | --- | --- | --- | --- | --- | --- | --- | --- | --- | --- | --- | --- | --- | --- | --- | --- | --- | --- | --- | --- | --- | --- | --- | --- | --- | --- | --- | --- | --- | --- | --- | --- |
| *Spyridia sp.* | 70 | 25 | 45 | 35 | 35 | 50 | 40 | 10 | 25 | 10 | 30 | 50 | 35 | 50 | 20 | 20 | 35 | 45 | 20 | 30 | 25 | 20 | 35 | 35 | 10 | 20 | 35 | 45 | 45 | 75 | 20 | 10 |
| *Ulva intestinalis* | 0 | 0 | 0 | 0 | 0 | 0 | 0 | 0 | 0 | 0 | 0 | 0 | 0 | 0 | 0 | 0 | 0 | 0 | 0 | 0 | 0 | 0 | 0 | 0 | 0 | 0 | 3 | 0 | 0 | 0 | 0 | 0 |
| *Dictyota* sp. | 10 | 0 | 1 | 0 | 0 | 5 | 3 | 1 | 1 | 0 | 3 | 3 | 1 | 0 | 0 | 0 | 0 | 1 | 0 | 1 | 0 | 3 | 0 | 0 | 0 | 0 | 0 | 0 | 0 | 0 | 1 | 0 |
| *Centroceras clavulatum* | 0 | 20 | 0 | 0 | 20 | 0 | 0 | 0 | 10 | 25 | 15 | 10 | 10 | 15 | 15 | 15 | 0 | 0 | 20 | 0 | 35 | 0 | 0 | 0 | 25 | 5 | 0 | 0 | 10 | 0 | 0 | 10 |
| *Sargassum* sp. | 0 | 1 | 1 | 1 | 1 | 1 | 0 | 1 | 1 | 0 | 0 | 0 | 1 | 1 | 0 | 0 | 5 | 1 | 1 | 1 | 5 | 0 | 1 | 1 | 1 | 1 | 0 | 0 | 3 | 1 | 0 | 1 |
| *Hypnea cervicornis* | 1 | 0 | 0 | 1 | 3 | 0 | 0 | 0 | 1 | 3 | 5 | 3 | 1 | 0 | 3 | 10 | 0 | 0 | 0 | 0 | 3 | 0 | 0 | 0 | 3 | 3 | 0 | 1 | 5 | 0 | 0 | 3 |
| *Ulva* sp. | 0 | 3 | 1 | 0 | 0 | 0 | 1 | 0 | 1 | 1 | 1 | 3 | 0 | 0 | 1 | 0 | 0 | 3 | 1 | 0 | 3 | 3 | 3 | 5 | 3 | 1 | 3 | 1 | 1 | 1 | 3 | 1 |
| *Padina gymnospora* | 3 | 3 | 3 | 1 | 0 | 0 | 1 | 0 | 0 | 0 | 0 | 0 | 0 | 0 | 0 | 0 | 0 | 1 | 0 | 0 | 0 | 0 | 1 | 0 | 0 | 0 | 1 | 0 | 0 | 1 | 1 | 0 |
| *Laurencia obtusa* | 0 | 0 | 3 | 0 | 1 | 0 | 0 | 0 | 1 | 0 | 0 | 0 | 0 | 1 | 0 | 0 | 0 | 0 | 5 | 0 | 0 | 0 | 0 | 5 | 1 | 1 | 2 | 0 | 1 | 0 | 0 | 1 |
| *Ceramium sp.* | 0 | 0 | 0 | 0 | 0 | 0 | 0 | 0 | 0 | 0 | 0 | 0 | 0 | 0 | 0 | 0 | 0 | 0 | 0 | 0 | 0 | 0 | 0 | 0 | 0 | 0 | 0 | 0 | 0 | 0 | 0 | 0 |
| Serpulidae | 10 | 20 | 10 | 10 | 10 | 3 | 5 | 20 | 20 | 20 | 15 | 10 | 5 | 5 | 20 | 25 | 20 | 15 | 20 | 40 | 20 | 40 | 15 | 15 | 15 | 10 | 10 | 5 | 15 | 10 | 25 | 15 |
| *Amphibalanus* sp. | 0 | 0 | 0 | 0 | 0 | 0 | 0 | 0 | 0 | 0 | 0 | 0 | 0 | 0 | 3 | 0 | 0 | 0 | 1 | 0 | 1 | 0 | 0 | 0 | 0 | 0 | 0 | 0 | 0 | 0 | 0 | 0 |
| Biofilm | 20 | 30 | 55 | 65 | 60 | 55 | 60 | 90 | 45 | 40 | 50 | 40 | 50 | 50 | 60 | 65 | 40 | 40 | 25 | 0 | 50 | 15 | 70 | 65 | 50 | 60 | 65 | 55 | 50 | 25 | 35 | 50 |
| Corallinaceae (CCA) | 1 | 10 | 0 | 0 | 0 | 1 | 0 | 1 | 3 | 3 | 0 | 3 | 1 | 1 | 3 | 0 | 3 | 1 | 3 | 0 | 1 | 1 | 1 | 3 | 1 | 0 | 3 | 0 | 0 | 0 | 1 | 5 |
| *Cladophora* sp. | 3 | 5 | 5 | 3 | 3 | 1 | 1 | 3 | 1 | 5 | 1 | 1 | 1 | 0 | 1 | 10 | 1 | 1 | 3 | 3 | 1 | 0 | 1 | 3 | 3 | 3 | 3 | 1 | 1 | 0 | 3 | 5 |
| Ectocarpaceae | 0 | 0 | 0 | 0 | 0 | 0 | 0 | 0 | 0 | 0 | 0 | 0 | 0 | 0 | 0 | 0 | 0 | 0 | 0 | 0 | 0 | 0 | 0 | 0 | 0 | 0 | 1 | 0 | 0 | 0 | 0 | 0 |
| *Jania adhaerens* | 5 | 1 | 5 | 0 | 0 | 0 | 0 | 1 | 0 | 0 | 0 | 0 | 5 | 1 | 5 | 5 | 0 | 0 | 1 | 0 | 1 | 0 | 0 | 0 | 3 | 1 | 10 | 3 | 0 | 0 | 0 | 1 |
| *Balanus trigonus* | 0 | 5 | 0 | 0 | 0 | 0 | 0 | 1 | 1 | 0 | 1 | 1 | 1 | 1 | 5 | 10 | 5 | 3 | 3 | 3 | 0 | 3 | 5 | 0 | 0 | 1 | 10 | 3 | 0 | 3 | 3 | 20 |
| Bryozoa 1 | 0 | 0 | 0 | 0 | 0 | 0 | 0 | 0 | 0 | 1 | 0 | 0 | 0 | 0 | 0 | 0 | 0 | 0 | 0 | 0 | 0 | 0 | 0 | 0 | 0 | 0 | 0 | 0 | 0 | 0 | 1 | 3 |
| Ascidia | 0 | 1 | 0 | 0 | 0 | 0 | 0 | 0 | 0 | 0 | 0 | 0 | 0 | 0 | 0 | 0 | 0 | 0 | 0 | 0 | 0 | 0 | 0 | 0 | 0 | 0 | 0 | 0 | 0 | 0 | 0 | 0 |
| *Schizoporella errata* | 0 | 0 | 0 | 0 | 0 | 0 | 0 | 0 | 0 | 0 | 10 | 0 | 0 | 0 | 0 | 0 | 0 | 0 | 0 | 0 | 0 | 0 | 0 | 0 | 0 | 0 | 0 | 0 | 0 | 0 | 0 | 0 |
| *Colpomenia sinuosa* | 0 | 0 | 0 | 0 | 0 | 0 | 0 | 0 | 0 | 0 | 0 | 0 | 0 | 0 | 0 | 0 | 0 | 0 | 0 | 0 | 0 | 0 | 0 | 0 | 0 | 0 | 0 | 0 | 0 | 0 | 0 | 1 |

Table 2. Live colonies recruitment experiment. Percent cover per replicate for treatment plates.

Table 3 Crude extract recruitment experiment. Percent cover per replicate for acrylic disks.

| Taxon | Control | Control | Control | Control | Control | Control | *P.dilatata* | *P.dilatata* | *P.dilatata* | *P.dilatata* | *P.dilatata* | *P.dilatata* |
| --- | --- | --- | --- | --- | --- | --- | --- | --- | --- | --- | --- | --- |
| *Cladophora* sp. | 1,3 | 1 | 0,3 | 1,7 | 2 | 1,3 | 3,3 | 2,7 | 2,3 | 1,3 | 0,7 | 1,3 |
| Ectocarpaceae | 7 | 16,7 | 32 | 23,7 | 19 | 10 | 8,7 | 29,3 | 28 | 28,7 | 38,7 | 37,3 |
| *Diatomeae* | 0,3 | 12,7 | 29,7 | 18 | 13,3 | 16,7 | 7,3 | 36,3 | 23 | 29,3 | 22 | 23,7 |
| *Obelia sp.* | 89 | 64 | 36,3 | 44,7 | 64,7 | 50 | 51,3 | 25,7 | 36,7 | 32,3 | 30 | 26,3 |
| *Balanus trigonus* | 0,7 | 0 | 0,3 | 2,3 | 0,7 | 0 | 0 | 2 | 4 | 1,3 | 3,7 | 4 |
| *Spyridia* sp. | 1 | 0 | 1 | 1,7 | 0 | 0 | 1 | 1,3 | 1 | 2 | 0,3 | 0,3 |
| *Ceramium sp.* | 0 | 0,3 | 0,3 | 0,7 | 0,3 | 0 | 1 | 0,3 | 1 | 0 | 1 | 3 |
| Corallinaceae (CCA) | 0 | 0 | 0 | 0,7 | 0 | 3,3 | 0 | 0,3 | 2 | 0,3 | 2,7 | 2,7 |
| *Membranipora membranacea* | 0 | 0 | 0 | 0 | 0 | 0 | 0 | 1 | 0 | 0 | 0 | 0,3 |
| *Cyanobacteria* | 0 | 0 | 0 | 2,3 | 0 | 0 | 0 | 1,7 | 1,7 | 0 | 1 | 0,3 |
| Ascidiacea | 0 | 0 | 0 | 0 | 0 | 0 | 0 | 0 | 0 | 0,3 | 0 | 0,3 |
| *Serpulidae* | 0 | 0 | 0 | 0,3 | 0 | 0 | 0 | 0 | 0 | 0 | 0 | 0,3 |
| *Colpomenia sinuosa* | 0 | 0 | 0 | 0 | 0 | 0 | 0 | 0 | 0 | 0,3 | 0 | 0 |

Table 4 Total area cover ( cm^2^ ) of *Palythoa caribaeorum* over difusion chambers .

| T1(Control) | T1(*P.dilatata*) | T2(Control) | T2(*P.dilatata*) | T3(Control) | T3(*P.dilatata*) |
| --- | --- | --- | --- | --- | --- |
| 4,287 | 1,337 | 8,064 | 5,414 | 2,876 | 1,311 |
| 4,528 | 2,85 | 5,831 | 3,419 | 4,527 | 4,263 |
| 5,752 | 1,071 | 5,394 | 3,458 | 5,463 | 4,365 |
| 0,313 | 1,141 | 1,58 | 0 | 4,872 | 2,736 |
| 2,176 | 2,023 | 3,601 | 0,321 | 3,957 | 1,943 |
| 3,008 | 0 | 6,979 | 0 | 2,862 | 0 |
