# Supplementary material for: Structuring effects of chemicals from the sea fan Phyllogorgia dilatata on benthic communities
Source: PeerJ. 2017 Apr 4;5:e3186. doi: 10.7717/peerj.3186 (PMC5382925; doi:10.7717/peerj.3186)
Supplement: Supplemental Information 3 — Table 1. Live colonies recruitment experiment. Percent cover per replicate for control plates near mimics. Table 2. Live colonies recruitment experiment. Percent cover per replicate for treatment plates near live gorgonians. Table 3. Crude extract recruitment experiment. Percent cover per replicate for acrylic disks. Table 4. Total area cover (cm2) of Palythoa caribaeorum on wells over time. [file peerj-05-3186-s003.pdf]

Table 1. Live colonies recruitment experiment. Percent cover per replicate for control plates near mimics.

	MIMIC CONTROL																															
	1A	1B	1C	1D	1E	1F	1G	1H	1I	1J	1L	1M	1N	1O	1P	1Q	3A	3B	3C	3D	3E	3F	3G	3H	3I	3J	3L	3M	3N	3O	3P	3Q
Spyridia sp.	5	15	10	5	35	25	45	20	25	15	25	20	20	20	35	20	1	20	10	0	45	20	15	20	25	20	15	5	35	30	25	20
Ulva intestinalis	0	0	0	0	0	0	0	0	0	0	0	0	0	0	0	0	0	0	0	0	0	0	0	0	0	0	0	0	0	0	0	0
Dictyota sp.	1	0	0	0	1	1	0	0	0	1	1	5	0	0	0	0	0	0	3	0	3	0	0	0	0	0	1	0	1	0	0	1
Centroceras clavulatum	0	5	3	0	3	10	1	10	10	10	15	5	1	0	1	15	0	1	0	0	0	0	30	20	0	0	0	1	0	0	0	0
Sargassum sp.	3	3	3	1	0	0	0	1	3	1	1	1	0	3	0	1	0	1	1	0	3	0	1	3	1	0	0	1	1	1	1	1
Hypnea cervicornis	3	5	3	1	1	3	1	3	3	1	5	5	1	0	3	10	1	0	1	1	0	0	10	5	0	1	1	1	1	0	1	0
Ulva sp.	3	3	10	5	0	0	0	1	1	3	1	1	3	1	3	5	5	3	1	3	1	1	3	1	5	3	3	3	3	3	5	3
Padina gymnospora	1	0	1	3	0	1	0	0	0	0	0	0	0	1	1	1	0	0	3	0	1	1	0	0	0	0	0	0	0	0	0	0
Laurencia obtusa	0	0	1	0	0	1	10	5	5	1	0	10	5	1	1	3	0	1	0	0	3	10	0	3	0	0	0	0	0	0	3	3
Ceramium sp.	0	0	0	0	0	0	0	0	0	0	0	0	0	0	0	0	0	0	0	0	0	0	0	0	0	0	0	0	0	0	0	0
Serpulidae	70	50	45	45	5	25	10	25	35	25	30	30	15	10	30	25	50	50	45	65	10	15	50	20	40	35	15	70	10	15	25	60
Amphibalanus sp.	1	1	5	0	1	0	0	0	1	1	3	0	0	5	0	0	0	0	0	0	0	0	3	0	0	0	0	0	0	0	0	0
Biofilm	10	5	10	0	65	30	40	30	40	45	20	35	0	60	15	15	0	3	3	15	75	90	30	20	35	15	0	5	60	60	45	3
Corallinaceae (CCA)	3	3	5	3	1	3	3	3	3	3	3	1	3	5	1	5	5	1	1	3	0	1	3	5	1	0	0	0	1	0	1	1
Cladophora sp.	0	5	5	1	1	3	1	0	0	1	1	1	3	3	1	10	3	3	3	3	3	3	3	3	1	3	3	1	3	3	0	1
Ectocarpaceae	0	0	0	0	0	0	0	0	0	0	0	0	0	0	0	0	0	0	0	0	0	0	0	0	0	0	0	0	0	0	0	0
Jania adhaerens	0	0	0	0	0	1	1	0	1	1	0	1	3	1	1	0	0	1	0	0	0	1	0	1	0	0	0	0	1	0	0	0
Balanus trigonus	3	3	3	10	1	1	5	1	0	3	1	0	5	0	3	1	1	0	5	5	0	0	3	0	0	5	5	10	0	5	0	3
Bryozoa 1	0	0	0	1	0	0	0	0	0	0	0	0	0	0	0	0	0	0	0	0	0	0	0	0	0	1	0	0	0	0	0	0
Ascidia	0	0	0	0	0	0	0	0	0	0	0	0	0	0	0	0	0	0	0	0	0	0	0	0	0	0	0	0	0	0	0	0
Schizoporella errata	0	0	0	0	0	0	0	0	0	0	0	0	0	0	0	0	0	0	0	0	0	0	0	0	0	0	0	0	0	0	0	0
Colpomenia sinuosa	0	0	0	0	0	0	0	0	0	0	0	0	0	0	0	1	0	0	0	0	0	0	0	0	0	0	0	0	0	0	0	0

Table 2. Live colonies recruitment experiment. Percent cover per replicate for treatment plates near live gorgonians.

	LIVE GORGONIANS TREATMENT																															
	2A	2B	2C	2D	2E	2F	2G	2H	2I	2J	2L	2M	2N	2O	2P	2Q	4A	4B	4C	4D	4E	4F	4G	4H	4I	4J	4L	4M	4N	4O	4P	4Q
Spyridia sp.	70	25	45	35	35	50	40	10	25	10	30	50	35	50	20	20	35	45	20	30	25	20	35	35	10	20	35	45	45	75	20	10
Ulva intestinalis	0	0	0	0	0	0	0	0	0	0	0	0	0	0	0	0	0	0	0	0	0	0	0	0	0	0	3	0	0	0	0	0
Dictyota sp.	10	0	1	0	0	5	3	1	1	0	3	3	1	0	0	0	0	1	0	1	0	3	0	0	0	0	0	0	0	0	1	0
Centroceras clavulatum	0	20	0	0	20	0	0	0	10	25	15	10	10	15	15	15	0	0	20	0	35	0	0	0	25	5	0	0	10	0	0	10
Sargassum sp.	0	1	1	1	1	1	0	1	1	0	0	0	1	1	0	0	5	1	1	1	5	0	1	1	1	1	0	0	3	1	0	1
Hypnea cervicornis	1	0	0	1	3	0	0	0	1	3	5	3	1	0	3	10	0	0	0	0	3	0	0	0	3	3	0	1	5	0	0	3
Ulva sp.	0	3	1	0	0	0	1	0	1	1	1	3	0	0	1	0	0	3	1	0	3	3	3	5	3	1	3	1	1	1	3	1
Padina gymnospora	3	3	3	1	0	0	1	0	0	0	0	0	0	0	0	0	0	1	0	0	0	0	1	0	0	0	1	0	0	1	1	0
Laurencia obtusa	0	0	3	0	1	0	0	0	1	0	0	0	0	1	0	0	0	0	5	0	0	0	0	5	1	1	2	0	1	0	0	1
Ceramium sp.	0	0	0	0	0	0	0	0	0	0	0	0	0	0	0	0	0	0	0	0	0	0	0	0	0	0	0	0	0	0	0	0
Serpulidae	10	20	10	10	10	3	5	20	20	20	15	10	5	5	20	25	20	15	20	40	20	40	15	15	15	10	10	5	15	10	25	15
Amphibalanus sp.	0	0	0	0	0	0	0	0	0	0	0	0	0	0	3	0	0	0	1	0	1	0	0	0	0	0	0	0	0	0	0	0
Biofilm	20	30	55	65	60	55	60	90	45	40	50	40	50	50	60	65	40	40	25	0	50	15	70	65	50	60	65	55	50	25	35	50
Corallinaceae (CCA)	1	10	0	0	0	1	0	1	3	3	0	3	1	1	3	0	3	1	3	0	1	1	1	3	1	0	3	0	0	0	1	5
Cladophora sp.	3	5	5	3	3	1	1	3	1	5	1	1	1	0	1	10	1	1	3	3	1	0	1	3	3	3	3	1	1	0	3	5
Ectocarpaceae	0	0	0	0	0	0	0	0	0	0	0	0	0	0	0	0	0	0	0	0	0	0	0	0	0	0	1	0	0	0	0	0
Jania adhaerens	5	1	5	0	0	0	0	1	0	0	0	0	5	1	5	5	0	0	1	0	1	0	0	0	3	1	10	3	0	0	0	1
Balanus trigonus	0	5	0	0	0	0	0	1	1	0	1	1	1	1	5	10	5	3	3	3	0	3	5	0	0	1	10	3	0	3	3	20
Bryozoa 1	0	0	0	0	0	0	0	0	0	1	0	0	0	0	0	0	0	0	0	0	0	0	0	0	0	0	0	0	0	0	1	3
Ascidia	0	1	0	0	0	0	0	0	0	0	0	0	0	0	0	0	0	0	0	0	0	0	0	0	0	0	0	0	0	0	0	0
Schizoporella errata	0	0	0	0	0	0	0	0	0	0	10	0	0	0	0	0	0	0	0	0	0	0	0	0	0	0	0	0	0	0	0	0
Colpomenia sinuosa	0	0	0	0	0	0	0	0	0	0	0	0	0	0	0	0	0	0	0	0	0	0	0	0	0	0	0	0	0	0	0	1

Table 3 Crude extract recruitment experiment. Percent cover per replicate for acrylic disks.

Taxon	Control	Control	Control	Control	Control	Control	*P.dilatata*	*P.dilatata*	*P.dilatata*	*P.dilatata*	*P.dilatata*	*P.dilatata*
Cladophora sp.	1.3	1	0.3	1.7	2	1.3	3.3	2.7	2.3	1.3	0.7	1.3
Ectocarpaceae	7	16.7	32	23.7	19	10	8.7	29.3	28	28.7	38.7	37.3
Diatomeae	0.3	12.7	29.7	18	13.3	16.7	7.3	36.3	23	29.3	22	23.7
Obelia sp.	89	64	36.3	44.7	64.7	50	51.3	25.7	36.7	32.3	30	26.3
Balanus trigonus	0.7	0	0.3	2.3	0.7	0	0	2	4	1.3	3.7	4
Spyridia sp.	1	0	1	1.7	0	0	1	1.3	1	2	0.3	0.3
Ceramium sp.	0	0.3	0.3	0.7	0.3	0	1	0.3	1	0	1	3
Corallinaceae (CCA)	0	0	0	0.7	0	3.3	0	0.3	2	0.3	2.7	2.7
Membranipora membranacea	0	0	0	0	0	0	0	1	0	0	0	0.3
Cyanobacteria	0	0	0	2.3	0	0	0	1.7	1.7	0	1	0.3
Ascidiacea	0	0	0	0	0	0	0	0	0	0.3	0	0.3
Serpulidae	0	0	0	0.3	0	0	0	0	0	0	0	0.3
Colpomenia sinuosa	0	0	0	0	0	0	0	0	0	0.3	0	0

Table 4 Total area cover (cm^2) of *Palythoa caribaeorum* on wells over time.

T1(Control)	T1(*P.dilatata*)	T2(Control)	T2(*P.dilatata*)	T3(Control)	T3(*P.dilatata*)
4.287	1.337	8.064	5.414	2.876	1.311
4.528	2.850	5.831	3.419	4.527	4.263
5.752	1.071	5.394	3.458	5.463	4.365
0.313	1.141	1.580	0	4.872	2.736
2.176	2.023	3.601	0.321	3.957	1.943
3.008	0	6.979	0	2.862	0
